# Primary Evaluation of Shape Recovery of Orthodontic Aligners Fabricated from Shape Memory Polymer (A Typodont Study)

**DOI:** 10.3390/dj9030031

**Published:** 2021-03-10

**Authors:** Tarek M. Elshazly, Ludger Keilig, Yasmine Alkabani, Ahmed Ghoneima, Moosa Abuzayda, Sameh Talaat, Christoph P. Bourauel

**Affiliations:** 1 Oral Technology Department, Dental School, University Hospital Bonn, Welschonnenstr. 17, 53111 Bonn, Germany; ludger.keilig@uni-bonn.de (L.K.); egyptortho@gmail.com (S.T.); bourauel@uni-bonn.de (C.P.B.); 2 Restorative and Dental Materials Department, National Research Centre, Giza 12622, Egypt; upper6@hotmail.com; 3 Department of Orthodontics, College of Dental Medicine, MBRU, P.O. Box 505055 Dubai, United Arab Emirates; ahmed.ghoneima@mbru.ac.ae; 4 Department of Prosthodontics, College of Dental Medicine, MBRU, P.O. Box 505055 Dubai, United Arab Emirates; Moosa.Abuzayda@mbru.ac.ae; 5 Department of Orthodontics, Future University in Egypt, Cairo 11835, Egypt

**Keywords:** orthodontics, smart polymers, clear aligners, orthodontic appliance, typodont, 3D printers, dentistry, digital workflow

## Abstract

As an innovative approach to overcome the rate-limiting staging of conventional aligners, using shape memory polymers (SMPs) as aligners’ materials was investigated in this in vitro study. The ability of SMPs to shape recover and consequently move tooth, upon appropriate stimuli, was evaluated on a typodont model before clinical application. The study design was to achieve 1.9 mm correction movement of an upper central incisor by one aligner after multiple steps/activation. A custom-made aligned typodont model with a movable upper central incisor was scanned. Using an orthodontic software and a 3D printer, resin-models were generated. Seven aligners of ClearX sheets (SMPs) were fabricated by thermoforming on the resin aligned model. Each aligner was tested for repositioning of the central incisor in the typodont model. The model was scanned after each step and the corrective movement was measured through the superimposition of scans. Results showed that the total correction efficiency of the SMPs’ aligner was ≈93% (1.76 mm). The corrective movement was 0.94 ± 0.04 mm after the reforming step, 0.66 ± 0.07 mm after the first activation step, and 0.15 ± 0.10 mm after the second activation step. It was concluded that aligners made of SMPs could have a promising future-use in orthodontic aesthetic treatment.

## 1. Introduction

Conventional orthodontic treatment by fixed appliances, such as braces and wires, is the backbone of orthodontic treatment. However, patients complain from many problems such as: mucosal irritation, soreness of the teeth, in addition to the difficulty of keep good oral hygiene. Moreover, due to the poor aesthetic appearance, some patients refuse having buccal braces, especially the adult ones [1,2,3]. Alternative treatment options have been introduced by many investigators to satisfy the high demand of an aesthetic orthodontic treatment; on top of these alternatives are ceramic or composite braces, lingual braces, and clear orthodontic aligners [4].

Clear aligners are a series of thin, clear, custom-made, removable, plastic aligners, which are created to effectively move teeth into their desired position. They should be worn for at least 20 h per day and changed approximately every two weeks [5]. Each aligner can limitedly move the teeth by around 0.2 to 0.3 mm for translations and 1° to 3° for rotations per tooth [6]. Clear aligners can be made of different polymers [7], such as polyethylene terephthalate glycol (PETG) [5] and polyurethane [8]. Clear orthodontic aligners have shown a reduced treatment period and shortened chair time in mild-to-moderate cases [9] and they have been proven to be an efficient and a feasible alternative to fixed braces [10,11]. However, still the rate-limiting staging of conventional aligners is limiting their use [5,6,9,12]. Therefore, investigators are working on improvements of aligner materials, force systems, staging of tooth movements, and treatment planning [13].

The aim of many recent interdisciplinary research studies is to introduce novel materials that can play an active role in the appliances [14]. These materials are called smart materials or stimuli-responsive materials which are able to react suitably with external stimuli, such as thermal, electrical, or magnetic input, producing a predictable repeatable output [15]. Shape memory materials are subcategory of smart materials which have the ability of changing their macroscopic shape upon a proper stimulus. Unlike shape changing materials, shape memory materials have the capacity to maintain a stable temporary shape until they are appropriately activated to recover their original shape [16,17].

Shape memory polymers (SMPs), also called actively moving polymers, are a type of smart shape memory material [17,18]. The shape memory mechanism of SMPs depends on presence of two pre-requites: a stable polymer network determines the original shape, and a reversible switching polymer responsible for fixing the temporary shape [19,20]. SMPs possess great attractiveness due to their significant elastic deformation ability, low cost, low density, ease of production, flexible programming, tailorable physical properties, excellent chemical stability, and high biocompatibility [21]. Because of these various advantages, SMPs may have great potential to penetrate virtually in several applications such as biomedical devices [22].

Specifically, thermo-responsive SMPs may have high potential as a novel orthodontic material, from functional and aesthetic point of views. Together with their relatively transparent and aesthetically satisfactory appearance, they have the advantage over the conventional aligner materials by possessing an intrinsic shape recovery property. Hence, application of SMPs to orthodontics can provide the aligners with considerable self-shape-recovery forces which may facilitate their operability and functionality [14,23].

In a primary study by Jung et al. [23], they used orthodontic wires made of shape memory polyurethane. After heating above transition temperature (50 °C), the teeth were corrected within one hour on a typodont model. Although there are some patents [24,25,26] that propose using smart polymers in fabrication of orthodontic aligners, there are still lack of studies investigating this innovation before being clinically applied [14].

The current study is a preliminary in vitro investigation of a type of orthodontic aligners made from thermal-responsive SMPs. The shape recovery forces generated upon appropriate thermal stimuli was used to move a tooth on a typodont. The aim was to overcome the rate-limiting staging of conventional aligner materials and show the possibility of using one shape memory aligner instead of three subsequent conventional aligners; in order to decrease the number of aligners used per treatment, saving money and time, reducing plastic consumption, and consequently decreasing the total cost.

## 2. Material and Methods

### 2.1. Specimens’ Preparation

Before establishing the following study design, a series of sensitivity tests were performed. Preliminary experiments were conducted to determine the best parameters especially with respect to temperature and pressure of deep-drawing (thermoforming) and reforming of the material sheets, as well as shape recovery. Furthermore, best parameters were determined for processing time which could end up in optimal results within the limitations of the used material. This means that every step of the following study design was preceded by several initial trials to adjust the optimal parameters that yield optimal results.

**Figure 1:**
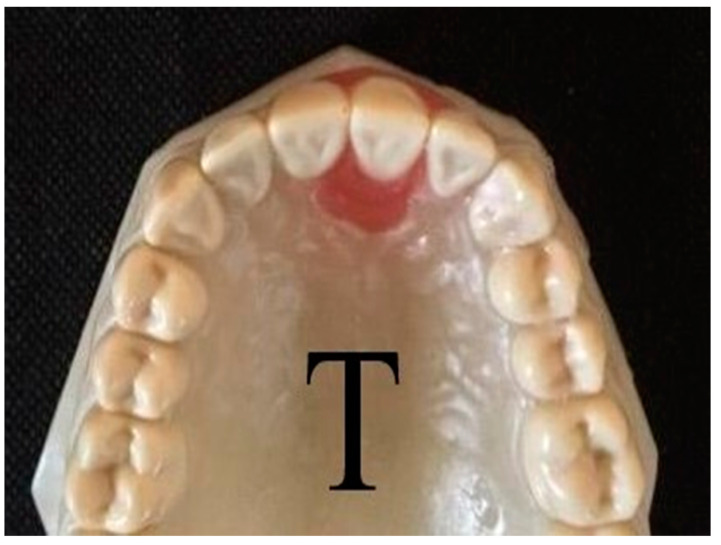
A custom-made typodont upper arch model with a movable left central incisor (Model T).

A custom-made typodont model (model T) was fabricated from acrylic teeth (Frasaco, Teltnag, Germany) and resin (Technovit 4004, Kulzer, Wehrheim, Germany). The movable acrylic upper left central incisor tooth was embedded in pink wax (Set up dental wax, Cavex, Harleem, the Netherlands), placed in the model, while the other typodont teeth were fixed by resin (Figure 1). The fully aligned model was then scanned (scan 0) using a 3D lab-scanner (D2000, 3Shape, Copenhagen, Denmark). After that, the model was segmented using Ortho System software (Ortho Analyzer v. 2012-1, 3Shape, Copenhagen, Denmark). Using the software, a palatal mal-alignment of 1.9 mm for the upper left central incisor tooth was designed, additionally, an intermediate model was also prepared with 1.2 mm mal-alignment (i.e., a correction of 0.7 mm) (Table 1).

**Figure 2:**
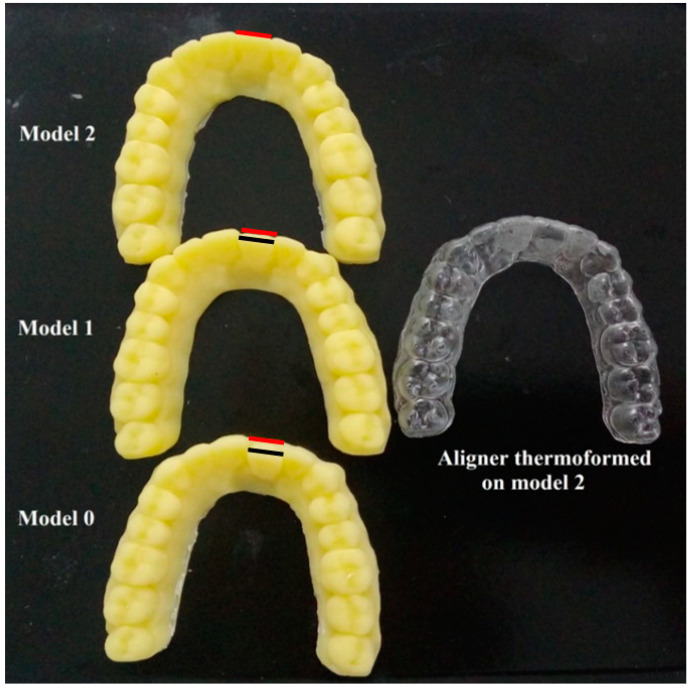
3D printed models with different mal-alignment of central incisor and a thermoformed aligner of ClearX material.

The three models (two mal-aligned models and one fully aligned model) were exported as STL files. The three models were 3D printed (Figure 2) using a printable resin (Dentona Optiprint model, Dentona AG, Dortmund, Germany) by a 3D Printer (Asiga Max, SCHEU-DENTAL GmbH, Iserlohn, Germany); model 0 (with 1.9 mm mal-alignment, i.e., 0.0 mm correction), model 1 (with 1.2 mm mal-alignment, i.e., 0.7 mm correction), and model 2 (with 0.0 mm mal-alignment, i.e., full 1.9 mm correction). A guiding splint was fabricated from a conventional thermoplastic sheet (Erkodur, Erkodent Erich Kopp, Pfalzgrafenweiler, Germany) with thickness 1.5 mm by thermopressing on model 0, and it was used as an index to ensure re-positioning of the movable typodont central incisor tooth in the same mal-aligned position before repeating the test, and it was made thick to ensure stiffness.

**Figure 3:**
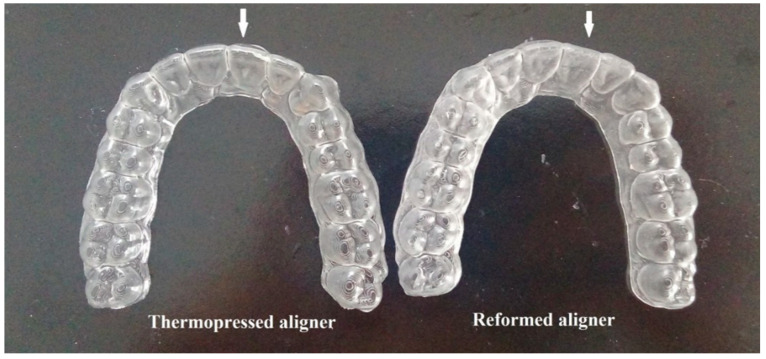
Thermopressed aligner on model 2 and reformed aligner on model 1.

In order to achieve a correction of 1.9 mm mal-alignment by only one aligner instead of three subsequent aligners, steps of thermoforming, reforming, and two activation cycles should be followed, respectively. A clear aligner was fabricated on the fully aligned model (model 2) using a shape memory sheet (ClearX) supplied by (Kline-Europe GmbH, Düsseldorf, Germany). The (0.76 mm thick) sheet was thermo-pressed by using a thermoforming device (Ministar, SCHEU-DENTAL GmbH, Iserlohn, Germany), by heating at 120 °C for 30 s, followed by pressing over the model at a 4 bar pressure (the recommended instructions provided by the supplier). Each aligner was then removed from the models, trimmed and finished. It was reformed on model 1 (with partial correction); where the reforming is a ClearX manufacturing step (introduced by Kline Europe, Düsseldorf, Germany) which is done to reshape the aligner on the previous step of the treatment plan (which has less tooth correction). Hence, in the current study, the thermoformed aligner on model 2 was reformed on the partially corrected model 1 (Figure 3). That was done by utilizing wet heating of the aligner in a warm water bath at 85 °C for 20 s followed by immediate pressure and heat adaptation on model 1 using the thermoforming device used earlier for the thermoforming step. So, basically, the aligner was heated once for the thermoforming step (to give it the original shape) and another time for the reforming step (to give it a temporary shape).

### 2.2. Testing of Shape Memory Correction on the Typodont Model

The wax around the upper left central incisor tooth in model T was softened. The guiding splint was used for positioning of the tooth in model T to be equally positioned as model 0. The model T was then placed in a 5 °C water bath for 10 min, to ensure that the wax is no longer soft and can withstand aligner placement without getting distorted [8]. Model T was then scanned (scan 1). The reformed aligner was then placed on model T, and together, they were placed in a hot water bath of 50 °C for 10 min. The model was placed on its base in a hot water basin and the water volume was adjusted to be at the level of the wax just below the aligner margin, to avoid, as much as possible, activation of the shape memory recovery of the aligner by the elevated temperature, meanwhile the wax was softened by hot water to allow the imbedded tooth to move. At the end, the model was replaced again in a water bath of 5 °C for 10 min to ensure wax hardening before aligner removal. After that, the model was scanned again (scan 2).

**Figure 4:**
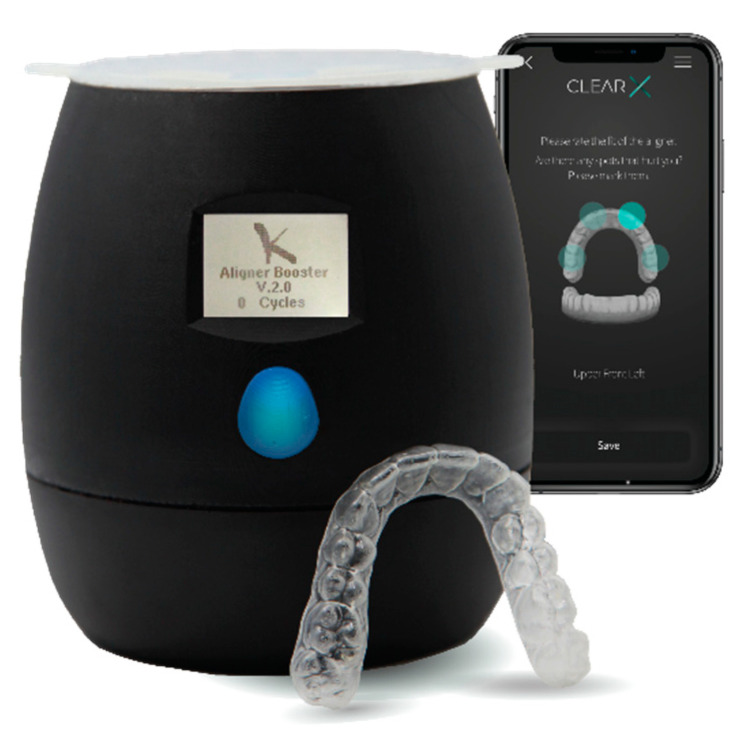
ClearX booster device controlled by a mobile application used for programmed activation of the ClearX aligners.

Afterwards, and to initiate the shape memory recovery, the aligner received the first activation cycle by placing it in an activation device (ClearX aligner booster v. 2.1, Kline-Europe GmbH, Düsseldorf, Germany). The ClearX aligner booster (Figure 4) is a programmed electric device, wirelessly connected and controlled by a mobile application (ClearX Mobil App. v. 1.1.4, Kline-Europe GmbH, Düsseldorf, Germany) (available on both Apple and Google store), was developed to provide the necessary medium for the shape memory aligner to regain its original shape through heating the aligner for a certain period of time in a hot water of certain temperature. The activation was done by keeping the aligner within the container of the device submerged in a hot water at 67 °C for 10 min. The activated aligner was then placed on model T, then they were placed together in a hot water bath of 50 °C for 10 min. Again, the water volume was adjusted to be at the level of the wax just below the aligner margin. After that, the model T was scanned (scan 3). Afterwards, the aligner received its second activation cycle, then the activated aligner was then placed on model T, and together, they were placed in a hot water bath of 50 °C for 10 min in the same way it was done before, and the model T was rescanned (scan 4).

This experiment was repeated 7 times, i.e., a total of 7 aligners were used in the study (*n* = 7), each time a new aligner was used to ensure the repeatability and the consistency of the results. Additionally, new wax was used each time to ensure that its properties were not altered by repeated heating and cooling cycles. In addition, the initial mal-aligned position was rescanned (scan 1), after using the guiding splint to re-position the typodont upper left central incisor tooth, and before using each new aligner to avoid any reading errors.

### 2.3. Analysis of Digital Models

**Figure 5:**
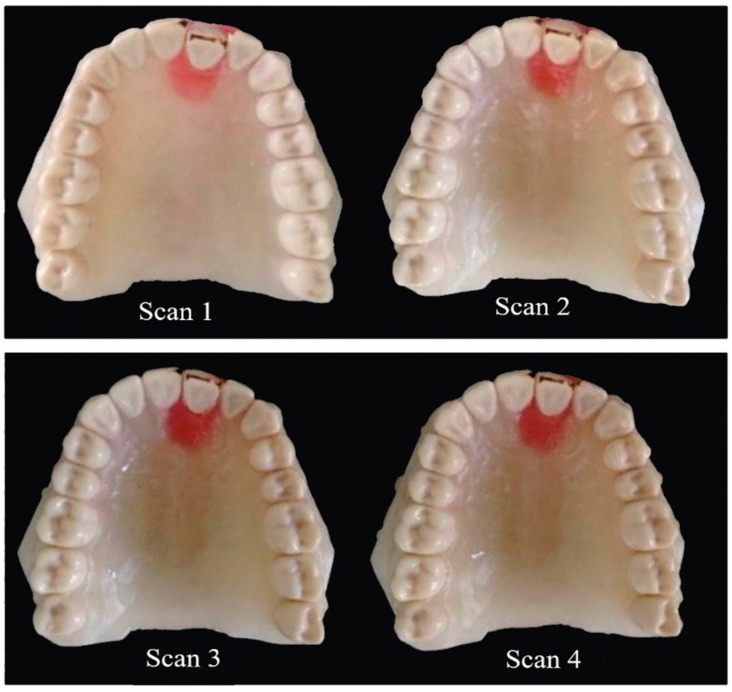
Model T before scanning at the different stages of treatment.

For each aligner, four 3D digital scans of Model T were obtained after different steps (Figure 5), which were: one scan after using the guiding splint for the initial 1.9 mm mal-aligned model (scan 1), a second scan after using the reformed aligner (scan 2), a third scan after using the aligner with first activation cycle (scan 3), and a fourth scan after using the aligner with second activation cycle (scan 4).

**Figure 6:**
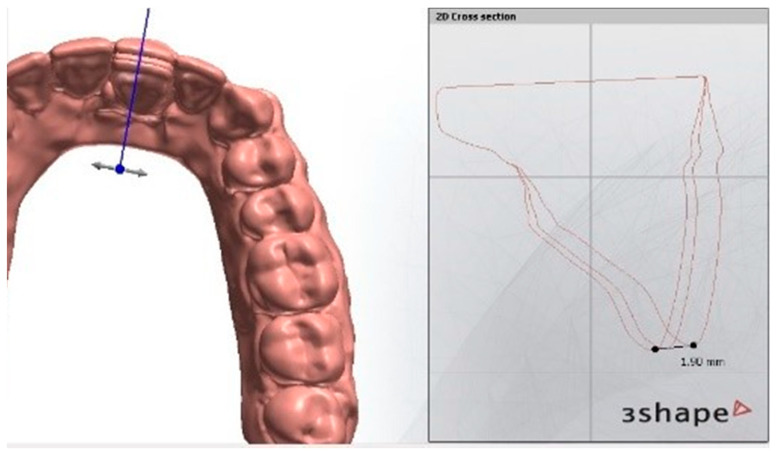
Superimposition of the typodont’s scans using 3Shape Ortho System software (Ortho Analyzer) and the amount of tooth movement was measured for each step.

**Figure 7:**
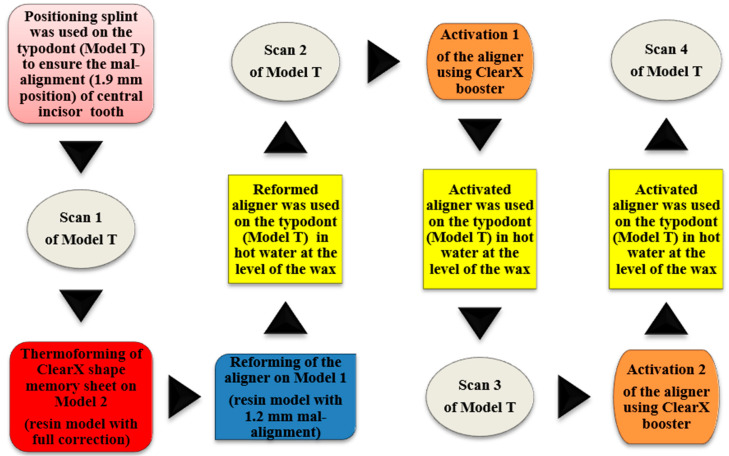
A schematic diagram illustrating the main steps of ClearX method.

The scans were superimposed using the Ortho Analyzer software, and the amount of tooth movement was measured in mm for each step, compared to the initial position in scan 1 and also compared to the position in the previous step (Figure 6). A list of all models and scans used in this study is illustrated in Table 1. A schematic diagram illustrating the main steps of ClearX method is shown in Figure 7.

## 3. Results

**Figure 8:**
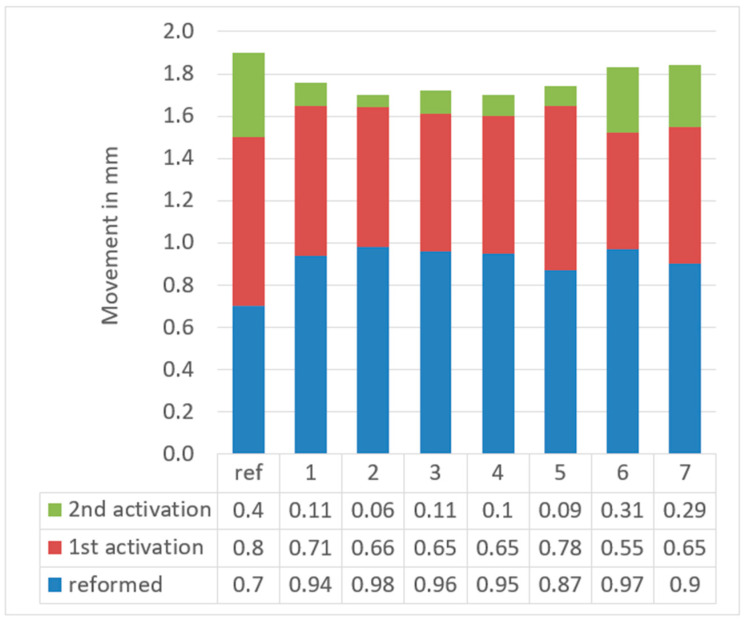
Amount of correction of the position of the upper left central incisor tooth on a typodont model per step by seven ClearX aligners.

Considerable corrective repositioning movements of the upper left central incisor tooth on the typodont were observed after each step. The added corrective movement after using the reformed aligner was 0.94 ± 0.04 mm to give an average correction of 49.47% of the total movement (scan 2 compared to scan 1), while after the first activation was 0.66 ± 0.07 mm to give an average added correction of 34.74% from the total planned movement (scan 3 compared to scan 2), and for the second activation was 0.15 ± 0.10 mm to give an average added correction of 7.89% of the total planned movement (scan 4 compared to scan 3). Results are shown in Table 2 and Table 3, as well as Figure 8.

## 4. Discussion

The aim of all optimizations, innovations, or advances in the orthodontic aligner field is mainly to facilitate the fabrication and treatment process, as well as to reduce time and cost of the treatment. The introduction of digital technology in fabrication of orthodontic aligners has been one of the most noteworthy orthodontic advances in this century [1,3]. Introduction of new materials for aligner fabrication pull the attention of some researchers [24,25,26]. It is always difficult when it comes to favor a suitable material, especially when it is a must to consider the biocompatibility and biomechanical behavior. Moreover, it was a challenge to introduce a clinically applicable method to evaluate the shape memory property of the material. For these reasons, full investigation of the used material and several in vitro studies should be done first before any clinical application. In the current study, it was proposed to use smart polymers in fabrication of orthodontics aligners, particularly thermo-responsive SMPs, which have the ability to keep two or more shapes and recover their permanent shape upon exposure to an appropriate thermal stimulus, or a series of stimuli [27].

ClearX system sheets, which were introduced by Kline Europe GmbH, are claimed to be a thermo-responsive shape memory polyurethane-based thermoplastic material. It is claimed that the material has the ability to recover to its original thermoformed shape after a reforming step, by a process of thermal activation at specific temperature for a certain period of time. Due to the stepwise shape changing property, this material was proposed to be used for the fabrication of orthodontic aligners, as it may successfully be used to overcome the rate-limiting staging of conventional aligners, in a way that one aligner may be able to replace three subsequent conventional aligners. Consequently, the number of aligners used per treatment could be reduced, beside saving money and time, especially for long and more complex therapies such as molar distalization and severe open/deep bite correction that are frequently performed nowadays [28,29,30].

Additionally, the method tested in the present investigation is easily linkable with CAD/CAM systems, which registered a constantly increasing use in many fields of dentistry, such as restorative dentistry, prosthodontics, and orthodontics. CAD/CAM technology allows a completely digital workflow, from impression to final framework, with clinical reliability [31] and good patients feedback [32].

It was found that nearly 92.63% of total correction efficiency could be reached on a typodont through one step of reforming and two steps of activation, i.e., three steps of treatment. The shape recovery behavior of the material is not only influenced by the chemical structure and composition of the polymer molecules, but also by the processing conditions. Controlling these conditions is important for controlling the properties of the material in practical applications [33]. Therefore, the whole study has been set up after performing a series of sensitivity tests. Several changeable parameters could control the result and determine the success of sufficient shape recovery. Temperature, moisture, and time of each step were the main governing parameters. Starting from the thermoforming step, passing by the reforming step, and ending with the activation step, all have showed high sensitivity. The activation of the shape memory property was done by the booster system (Figure 4). The appropriate parameters, required to accomplish the optimal shape recovery of the material by using the booster, were reached, after the sensitivity testing, in a way that the first activation cycle was found to initiate an average of 65% shape memory recovery in the aligner and the second cycle was found to initiate an average of 35% shape memory recovery.

The fundamental mechanism of shape memory effect in SMPs is the presence of a two-domain system with two different glass transition/melting temperatures. Hence, at an ambient temperature, one domain is being hard/elastic, while the other domain is soft/ductile [34]. In other words, the shape memory mechanism in thermal-responsive SMPs is a reversible activation and inactivation of polymer-chain motion in the switching segments respectively above and below certain temperature called transition temperature (T_trans_). T_trans_ could be either glass transition temperature (T_g_) or melting temperature (T_m_) [14,20,35]. So, once the T_trans_ is reached, the deformed shape memory material displays an elastic property and return to its original shape; this shape recovery generates forces that may be able to move a tooth [36].

SMPs could have more than one temporary shape, because they have a wider shape recovery temperature range and a much higher recoverable strain [19,27]. Shape memory polyurethane resins consist of both polar and non-polar molecules which segregate into micro domains of hard and soft segments. By combining hard and soft molecular domains, the material could achieve both high strength (from the hard regions) and high toughness (from the soft regions), in a way that enables fabrication of durable orthodontic aligners which can move the tooth over longer period of time [33,37]. Additionally, the polyurethane resin is resistant to accumulation of deposits and stains, allowing it to stay clean in oral conditions for longer time, however, it shows sensitivity to moisture due to presence of hydrogen bonding [37,38,39]. That could explain why the moisture was a governing factor at the reforming and activation steps of ClearX aligners.

Ideally speaking, the reformed aligner should make a 0.7 mm corrective movement, while the first activation should result in an average of 55–65% shape recovery, and the second activation should result in an average 25–35% recovery, depending on the type and amount of the planned movement. However, the results of the present study showed an average movement of 0.94 mm for the reformed aligners, which is higher than the planned 0.7 mm movement (Table 2, and Figure 8). This could be attributed to a spontaneous recovery occurring during stress release, or, could be in part triggered by a partial shape memory recovery caused by the heat generated by the 50 °C water bath used in this study to soften the wax. Thus, an average of 0.24 mm extra movement was achieved by the reformed aligner (20% of the total shape memory component of the movement).

Upon first activation, the aligners gave an average 0.66 mm added movement, which compromise 55% of the planned shaped memory movement, and thus give an average total movement of 1.6 mm (84.2% of the total planned movement of 1.9 mm). The second activation gave an average added movement of 0.15 mm which corresponds to 12.5% of the total planned shape memory movement, and thus giving an average total movement of 1.75 mm (92.63% of the total planned movement).

In the ClearX system, the manufacturer claimed that the slight unrecovered residual part of movement (≈7–8%) should be achieved using the next aligner before activation, which the company refers to as recurrent aligner, this also gives another chance to any lagging orthodontic movement. Thus, the next aligner should be first used to confirm that full movement of the previous aligner is achieved and then it is activated to deliver additional movements, and so on.

The comparable results between specimens showed a consistent behavior of the material. Although the results are promising, this study could only be considered as a proof of concept and it still has many limitations. It is just a preliminary in vitro study on a typodont. In the typodont studies, the wax substitutes the periodontium. However, the bone modeling due to orthodontic forces is a complicated biological process resulting from a complex biomechanical reaction of the biological tissues of the periodontium [40]. Additionally, an idealized movable single tooth movement was conducted, while the other teeth were fixed in the resin of the typodont model, yet, such situation is not clinically related. Moreover, within the range of activations’ temperature, it was neglected that in the clinical situation, the hot foods and/or drinks can affect the whole treatment process, as they can activate the aligner in between and distort the whole design of treatment. Furthermore, the mechanical behavior of the aligner should be thoroughly studied and the delivered forces by the shape memory recovery should be measured. Such investigations and limitations will be considered in further upcoming studies.

## 5. Conclusions

Experimentally, tooth movement could be conducted on a typodont model by using clear aligners made of shape memory polymers (SMPs). The aligner, however, should undergo different steps of special heat treatment above its transition temperature in order to initiate its shape memory recovery. Consequently, aligners made of SMPs could be a promising future choice for orthodontic aesthetic treatment.

## Figures and Tables

**Table 1:** A list of models and scans used in the study.

Type	Name	Description	Use
**Models**	Model T	The typodont.	Scanned with fully aligned teeth (Scan 0, which was used for software manipulation). The movable central incisor tooth was used for showing the amount of movement achieved by the shape memory recovery of the aligner.
	Model 0	The 3D printed resin model with full mal-alignment (1.9 mm)	Used for fabrication of a guiding splint used for repositioning of the typodont movable central incisor tooth to the zero position.
	Model 1	The 3D printed resin model with partial mal-alignment (1.2 mm), i.e., partial correction (0.7 mm)	Used for reforming of the aligners.
	Model 2	The 3D printed resin model with full correction (1.9 mm)	Used for thermoforming of the aligners.
**Scans**	Scan 0	A scan of the fully aligned typodont model.	Used for software manipulation and production of models 0, 1, and 2.
	Scan 1	A scan of the typodont model after using the guiding splint to move the central incisor tooth to the fully mal-aligned position.	Ideally should correspond to Model 0 shape.
	Scan 2	A scan of the typodont model after the movement of the central incisor tooth by using the reformed aligner.	Used for superimposition of the scans and measurement of amount of the tooth movement.
	Scan 3	A scan of the typodont model after the movement of the central incisor tooth by using the activated aligner received the first activation cycle.	Used for superimposition of the scans and measurement of amount of the tooth movement.
	Scan 4	A scan of the typodont model after the movement of the central incisor tooth by using the activated aligner received the second activation cycle.	Used for superimposition of the scans and measurement of amount of the tooth movement.

**Table 2:** Mean and standard deviation (SD) of the total cumulative correction (TC) of the position of the upper left central incisor tooth after superimposition of each step’s scan over the scan of the initial mal-position, the added correction (AC) after superimposition of each step’s scan over its predecessor step’s scan, and the percentage of correction efficiency by the ClearX aligner.

	Scan 1 vs. 2		Scan 1 vs. 3		Scan 1 vs. 4	
	TC	AC	TC	AC	TC	AC
Planned movement	0.70	0.70	1.50	0.80	1.90	0.40
Aligner 1	0.94	0.94	1.65	0.71	1.76	0.11
Aligner 2	0.98	0.98	1.64	0.66	1.70	0.06
Aligner 3	0.96	0.96	1.61	0.65	1.72	0.11
Aligner 4	0.95	0.95	1.60	0.65	1.70	0.10
Aligner 5	0.87	0.87	1.65	0.78	1.74	0.09
Aligner 6	0.97	0.97	1.52	0.55	1.83	0.31
Aligner 7	0.90	0.90	1.55	0.65	1.84	0.29
Mean (mm)	0.94	0.94	1.60	0.66	1.76	0.15
SD	0.04	0.04	0.07	0.07	0.10	0.10
Correction % (divided by 1.9 mm total movement)	49.47%	49.47%	84.21%	34.74%	92.63%	7.59%

**Table 3:** Numerical values and percentages of shape memory recovery components.

Recovery	Added Movement	% Recovery of Shape Memory Component Per Step (Divided by Total 1.2 mm)	% Recovery of Activated Shape Memory Component Per Step (Divided by Total 0.96 mm)
**Spontaneous**	0.24 mm	20%	
**First activation**	0.66 mm	55%	68.75%
**Second activation**	0.15 mm	12.5%	15.63%

## Data Availability

The data is available for the editor on request.

## Availability of Data and Material

Available on request.
